# Surveillance and Testing for Middle East Respiratory Syndrome Coronavirus, Saudi Arabia, April 2015–February 2016

**DOI:** 10.3201/eid2304.161793

**Published:** 2017-04

**Authors:** Abdulaziz A. Bin Saeed, Glen R. Abedi, Abdullah G. Alzahrani, Iyad Salameh, Fatima Abdirizak, Raafat Alhakeem, Homoud Algarni, Osman A. El Nil, Mutaz Mohammed, Abdullah M. Assiri, Hail M. Alabdely, John T. Watson, Susan I. Gerber

**Affiliations:** Ministry of Health, Riyadh, Saudi Arabia (A.A. Bin Saeed, A.G. Alzahrani, I. Salameh, R. Alhakeem, H. Algarni, O.A. El Nil, M. Mohammed, A.M. Assiri, H.M. Alabdely);; Centers for Disease Control and Prevention, Atlanta, Georgia, USA (G.R. Abedi, F. Abdirizak, J.T. Watson, S.I. Gerber).

**Keywords:** surveillance, testing, respiratory infections, Middle East respiratory syndrome coronavirus, MERS-CoV, Saudi Arabia, viruses, zoonoses, surveillance practices, infection control, transmission

## Abstract

Saudi Arabia has reported >80% of the Middle East respiratory syndrome coronavirus (MERS-CoV) cases worldwide. During April 2015–February 2016, Saudi Arabia identified and tested 57,363 persons (18.4/10,000 residents) with suspected MERS-CoV infection; 384 (0.7%) tested positive. Robust, extensive, and timely surveillance is critical for limiting virus transmission.

Middle East respiratory syndrome (MERS) coronavirus (CoV) causes severe respiratory illness in humans, with death occurring in >35% of reported cases (*1*). MERS has been documented among persons with close contact with known case-patients in healthcare (*2*) and household (*3*) settings and among persons with recent contact with dromedaries (*4*). Proper clinical management of persons with suspected MERS-CoV infection who seek care in a healthcare setting relies upon adherence to recommended infection-control precautions (*5*), which in turn depends upon the early recognition of cases.

The International Health Regulations Emergency Committee of the World Health Organization reported that data sharing for this disease, including sharing of surveillance results, “remains limited and has fallen short of expectations” (*6*). To determine the extent of MERS surveillance in Saudi Arabia, we reviewed electronic surveillance data collected during April 1, 2015–February 1, 2016, to describe trends in surveillance for MERS and to compare demographic and clinical features among persons tested.

## The Study

In Saudi Arabia, persons who should be tested for MERS-CoV include suspect case-patients who meet at least 1 of 4 case definition categories (Technical Appendix Table). In brief, the categories are persons with community-acquired pneumonia (category I); healthcare-associated pneumonia (II); symptoms after exposure to a MERS-CoV case-patient (III); or unexplained febrile illness (IV). The case definition was revised in May 2014 (*7*); additional refinements were made in June 2015 (*8*). The 2015 definition included changes to the approach for testing children <14 years of age with nonsevere illness (testing is reserved for children with exposure to camels or camel products or to a confirmed or suspected MERS case-patient). In addition to suspected cases, testing is recommended for close contacts of persons with confirmed MERS-CoV infection, regardless of symptoms, and can also be requested at the discretion of an infectious disease consultant. Tests are performed on respiratory specimens at 5 regional laboratories using real-time PCR (*9*).

**Figure 1:**
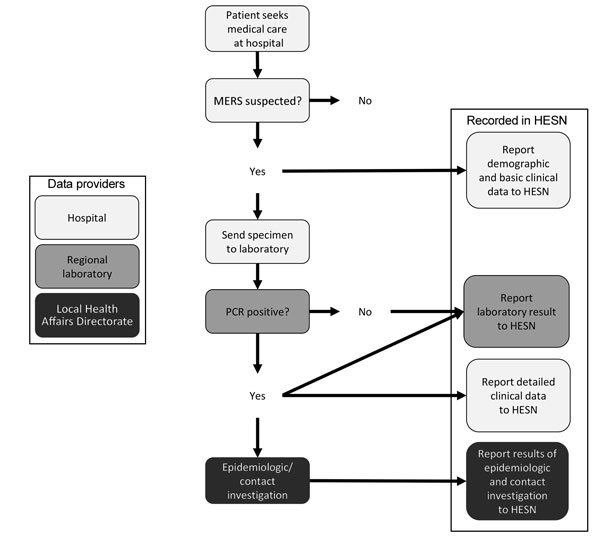
Reporting pathway for data regarding persons tested for Middle East respiratory syndrome coronavirus infection to the Health Electronic Surveillance Network (HESN), Saudi Arabia, 2014–2016.

Since March 7, 2015, official reporting of cases referred for MERS-CoV testing in Saudi Arabia has exclusively been documented through the Health Electronic Surveillance Network (HESN). When a suspected case-patient is identified for testing, the referring hospital reports demographic and basic clinical data to HESN (Figure 1). After specimens are submitted and testing completed, the regional laboratory reports the result to HESN. For positive cases, the referring hospital submits additional clinical information, and the local Health Affairs Directorate (HAD) initiates an investigation of exposures and contacts. For negative test results, no further action is taken in HESN. Surveillance activities occur in each of the 20 local HADs and among Hajj pilgrims. We analyzed demographic, clinical, and laboratory data for persons reported to HESN during April 1, 2015–February 1, 2016, in aggregate and by HAD using Microsoft Excel 2013 (Microsoft Corp., Redmond, WA, USA) and SAS version 9.3 (SAS Institute, Inc., Cary, NC, USA).

**Figure 2:**
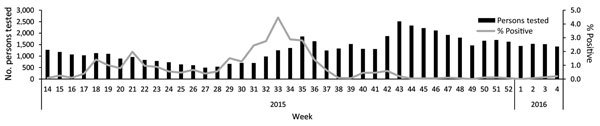
Number persons tested and percent positive for Middle East respiratory syndrome coronavirus, Saudi Arabia, April 1, 2015–February 1, 2016.

A total of 57,363 suspected MERS case-patients were identified and tested during the study period; 384 (0.7%) tested positive (Table 1). Among those for whom nationality and sex were known, 70.3% were Saudi (compared with 67.3% of the general population) and 54.3% were male. Rates of positivity among those with known age differed by age group; highest and lowest rates were among persons 50–65 and <14 years of age, respectively (Table 1). The month with the highest number of tested persons was November 2015 (n = 9,197) (Figure 2), and the month with the highest percentage of positive tested persons was August 2015 (3.4% of 4,770 tested persons).

**Table 1:** Demographic characteristics of persons tested for Middle East respiratory syndrome coronavirus, Saudi Arabia, April 1, 2015–February 1, 2016

Characteristic	No. (%) confirmed	No. (%) not confirmed	No. (%) total	% Positive
Overall	384	56,979 (99.3)	57,363 (100.0)	0.7
Sex				
F	156	25,863 (45.7)	26,019 (45.7)	0.6
M	226	30,718 (54.3)	30,944 (54.3)	0.7
Total	382	56,581	56,963	0.7
Nationality				
Saudi	246	34,628 (70.3)	34,874 (70.3)	0.7
Non Saudi	107	14,604 (29.7)	14,711 (29.7)	0.7
Total	353	49,232	49,585	0.7
Reason for testing				
Suspected case*	286	47,592 (89.1)	47,878 (89.0)	0.6
Category I	87	32,284 (60.5)	32,371 (60.2)	0.3
Category II	67	2,662 (5.0)	2,729 (5.1)	2.5
Category III	107	4,669 (8.7)	4,776 (8.9)	2.2
Category IV	25	7,977 (14.9)	8,002 (14.9)	0.3
Recommended by infectious disease consultant	66	3,256 (6.1)	3,322 (6.2)	2.0
Patient asymptomatic	19	2,555 (4.8)	2,574 (4.8)	0.7
Total	371	53,403	53,774	0.7
Month of report				
April 2015	10	4,953 (8.7)	4,963 (8.7)	0.2
May 2015	54	4,414 (7.7)	4,468 (7.8)	1.2
June 2015	24	3,090 (5.4)	3,114 (5.4)	0.8
July 2015	24	2,634 (4.6)	2,658 (4.6)	0.9
August 2015	160	4,610 (8.1)	4,770 (8.3)	3.4
September 2015	66	6,520 (11.4)	6,586 (11.5)	1.0
October 2015	28	7,568 (13.3)	7,596 (13.2)	0.4
November 2015	6	9,191 (16.1)	9,197 (16.0)	0.1
December 2015	5	7,280 (12.8)	7,285 (12.7)	0.1
January 2016	6	6,487 (11.4)	6,493 (11.3)	0.1
February 2016	1	232 (0.4)	233 (0.4)	0.4
Total	384	56,979	57,363	0.7
Age, y				
0–14	10	8,022 (14.2)	8,032 (14.1)	0.1
15–34	97	17,621 (31.1)	17,718 (31.1)	0.5
35–49	82	10,201 (18.0)	10,283 (18.0)	0.8
50–65	109	10,082 (17.8)	10,191 (17.9)	1.1
>66	84	10,692 (18.9)	10,776 (18.9)	0.8
Total	382	56,618	57,000	

*Categories: I, acute respiratory illness with clinical and/or radiologic evidence of pulmonary parenchymal disease (pneumonia or acute respiratory distress syndrome); II, a hospitalized patient with healthcare associated pneumonia based on clinical and radiological evidence; III, upper or lower respiratory illness within 2 weeks after exposure to a confirmed or probable case of MERS-CoV; IV, unexplained acute febrile (>38°C) illness, and body aches, headache, diarrhea, or nausea/vomiting, with or without respiratory symptoms, and leukopenia (white blood cell count <3.5 × 10^9^/L) and thrombocytopenia (platelets <150 × 10^9^/L). Descriptions are from the Saudi Arabia Ministry of Health MERS-CoV Case Definition and Surveillance Guidance–Updated June 2015 (http://www.moh.gov.sa/en/CCC/Regulations/Case%20Definition.pdf).

Among tested persons for whom the reason for testing was known, 89.0% met the clinical case definition for suspected MERS (Table 1). The remaining 11.0% were those recommended for testing by an infectious disease consultant and asymptomatic contacts of confirmed case-patients. More than half of those tested (60.2%) met the category I definition (community-acquired pneumonia) for a suspected case-patient; 0.3% tested positive. The highest positivity rate, 2.5%, was among persons meeting the category II definition for a suspected case-patient (healthcare-associated pneumonia). Among all persons with presumed exposure to MERS case-patients (persons meeting categories II, III and asymptomatic contacts of confirmed case-patients), the positivity rate was 1.9%. Among the 185 confirmed case-patients with available occupational information, 35 (18.9%) were healthcare workers (data not shown).

Most tested persons were reported in the course of routine surveillance through a local HAD. Nationwide, 18.4 persons/10,000 inhabitants were tested, and 1.2/100,000 were MERS-CoV–positive (*10*) (Table 2). Rates of testing and positivity varied by HAD; the highest testing rates were in Ahsa HAD, followed by Riyadh HAD. Najran HAD had the highest percentage of positive persons (Table 2).

**Table 2:** Middle East respiratory syndrome cases by local Health Affairs Directorate and among Hajj pilgrims, Saudi Arabia, April 1, 2015–February 1, 2016*

Surveillance population	Population	No. positive/no. tested	No. tested/10,000 population	No. confirmed cases/100,000 population	% Positive
Local Health Affairs Directorate					
Riyadh	7,717,467	276/22,322	28.9	3.6	1.2
Jeddah	4,224,568	10/6,606	15.6	0.2	0.2
Eastern	3,019,461	2/7,319	24.2	0.1	0
Makkah	2,111,127	2/3,729	17.7	0.1	0.1
Madinah	2,012,749	8/2,258	11.2	0.4	0.4
Asir	1,766,212	6/1,730	9.8	0.3	0.3
Jazan	1,533,496	0/790	5.2	0	0
Qasim	1,370,727	5/1,091	8.0	0.4	0.5
Taif	1,257,888	7/1,866	14.8	0.6	0.4
Ahsa	1,193,373	57/5,359	44.9	4.8	1.1
Tabuk	887,383	1/732	8.2	0.1	0.1
Hail	670,468	0/685	10.2	0	0
Najran	568,631	10/494	8.7	1.8	2.0
Baha	461,360	0/184	4.0	0	0
Hafr al-Batin	437,349	0/145	3.3	0	0
Bisha	379,521	0/227	6.0	0	0
Northern Borders	359,297	0/240	6.7	0	0
Jauf	329,277	0/390	11.8	0	0
Qunfudha	304,392	0/183	6.0	0	0
Qurayat	165,629	0/122	7.4	0	0
Total	30,770,375	384/56,472	18.4	1.2	0.7
Hajj pilgrims					
Total	1,952,817	0/888	4.5	0	0

*Population data from (*10*).

In addition, surveillance during the annual Hajj pilgrimage included 888 tested persons during September 2015, representing 4.5 tested persons/10,000 among 1,952,817 pilgrims. None tested positive for MERS-CoV.

Among 8,032 children <14 years of age, 10 (0.1%) tested positive, including 5 who were <1 year of age. At least 7 of the 10 children were tested because of exposure to a MERS case-patient. The number of tests among children <14 years of age temporarily dropped after the case definition revision in June 2015, which introduced more stringent criteria for testing.

## Conclusions

Surveillance and testing for MERS-CoV infection is extensive and widespread in Saudi Arabia. During our study, an average of >5,000 persons per month were identified as being at high risk for infection due to clinical or epidemiologic criteria and were subsequently tested. MERS was first recognized in 2012, and as of November 3, 2016, Saudi Arabia has reported 80.9% of the cases reported worldwide (*11*); this distinction may be partly due to the country’s robust implementation of surveillance practices and the ready availability of testing, which is facilitated by HESN. We found few other published descriptions of surveillance practices for MERS-CoV (*12*,*13*).

Confirmed MERS case-patients represented <1% of all tested persons in Saudi Arabia. Most tests were conducted for persons with community-acquired pneumonia, among whom the positivity rate was predictably low. Positivity rates were highest among persons tested because of presumed exposure to MERS case-patients (i.e., those tested because of healthcare-acquired pneumonia or onset of symptoms following contact with a confirmed case-patient).

Only 0.1% of children <14 years of age tested positive for MERS-CoV; this was the lowest rate among all age groups. Most MERS-CoV–positive children <14 years of age were tested because of high-risk exposures, not because they met clinical criteria. Although the proportion of positive tests was highest among persons >35 years of age, the number of tests was highest among persons 18–34 years of age, perhaps because of widespread testing of healthcare workers during outbreaks.

The largest number of tests was conducted in November, coinciding with the winter respiratory virus season. In comparison, the proportion of positive tests peaked in May and August, coinciding with outbreaks that occurred in Ahsa (*14*) and Riyadh (*15*), respectively.

Our analysis had limitations. Variations were probably present in the reporting practices of the various data reporters, in the clinical diagnostic practices used across Saudi Arabia, and among investigation teams. Such variations could affect the completeness, accuracy, and timeliness of the data used for this assessment.

Surveillance and testing for MERS-CoV throughout Saudi Arabia is extensive, as documented by HESN; in a single month during this study, >9,000 patients at high risk for MERS were investigated. A continued robust approach to the early detection of patients with MERS is critical for the prompt implementation of infection-control precautions and the prevention of healthcare-associated transmission of MERS-CoV.

Technical Appendix Definitions for suspected cases of Middle East respiratory syndrome coronavirus in Saudi Arabia.
