# Supplementary material for: Surveillance and Testing for Middle East Respiratory Syndrome Coronavirus, Saudi Arabia, April 2015–February 2016
Source: Emerg Infect Dis. 2017 Apr;23(4):682–5. doi: 10.3201/eid2304.161793 (PMC5367404; doi:10.3201/eid2304.161793)
Supplement: Technical Appendix — Definitions for suspected cases of Middle East respiratory syndrome coronavirus in Saudi Arabia. [file 16-1793-Techapp-s1.pdf]

Article DOI: http://dx.doi.org/10.3201/eid2304.161793

# Surveillance and Testing for Middle East Respiratory Syndrome Coronavirus, Saudi Arabia, April 2015–February 2016

## Technical Appendix

**Technical Appendix Table.** Definitions for suspected cases of Middle East Respiratory Syndrome Coronavirus in Saudi Arabia, published May 2014 and revised June 2015

| Date, patient age, category | Definition |
|---|---|
| June 2015* | |
| Adults (>14 y of age) | |
| I | Acute respiratory illness with clinical and/or radiological, evidence of pulmonary parenchymal disease (pneumonia or Acute Respiratory Distress Syndrome) |
| II | A hospitalized patient with healthcare associated pneumonia based on clinical and radiological evidence. |
| III | Upper or lower respiratory illness within 2 weeks after exposure to a confirmed or probable case of MERS-CoV infection. |
| IV | Unexplained acute febrile (≥38°C) illness, AND body aches, headache, diarrhea, or nausea/vomiting, with or without respiratory symptoms, AND leucopenia (leukocyte<3.5x109/L) and thrombocytopenia (platelets<150x109/L). |
| Children (≤14 y of age) | |
| I | Meets the above case definitions and has at least one of the following<br>a. History of exposure to a confirmed or suspected MERS CoV in the 14 d before onset of symptoms<br>b. History of contact with camels or camel products in the 14 d before onset of symptoms |
| II | Unexplained severe pneumonia |
| May 2014† | |
| All ages | |
| I | A person with fever and community-acquired pneumonia or acute respiratory distress syndrome based on clinical or radiological evidence. |
| II | A hospitalized patient with healthcare associated pneumonia based on clinical and radiological evidence. |
| III | A person with 1) acute febrile (≥38°C) illness, AND 2) body aches, headache, diarrhea, or nausea/vomiting, with or without respiratory symptoms, AND 3) unexplained leucopenia (leukocyte<3.5x109/L) and thrombocytopenia (platelets<150x109/L). |
| IV | A person (including health care workers) who had protected or unprotected exposure to a confirmed or probable case of MERS-CoV infection and who presents with upper or lower respiratory illness within 2 weeks after exposure. |

*http://www.moh.gov.sa/en/CCC/Regulations/Case%20Definition.pdf
†http://www.moh.gov.sa/en/Documents/MoHCaseDefinitionMERSCoVVersionMay132014.pdf
